# THE ASSOCIATION OF AIR POLLUTION AND CLIMATE FACTORS WITH DEPRESSIVE SYMPTOMS AMONG OLDER MEXICAN AMERICAN ADULTS

**DOI:** 10.1093/geroni/igad104.3793

**Published:** 2023-12-21

**Authors:** Diane Saab, Phillip Cantu, Brian Downer, Kyriakos Markides, Sadaf Milani

**Affiliations:** University of Texas Medical Branch, Galveston, Texas, United States; University of Texas Medical Branch, Galveston, Texas, United States; University of Texas Medical Branch, Galveston, Texas, United States; University of Texas Medical Branch, Galveston, Texas, United States; University of Texas Medical Branch, Galveston, Texas, United States

## Abstract

Air pollution, drought, and extreme heat are associated with elevated depressive symptoms. Older Mexican American adults experience high levels of depressive symptoms compared to other demographic groups, but little is known about the role of environmental factors in depressive symptoms for this population. Mexican American adults are resilient to the harmful health effects of socioeconomic disadvantage, but it is unclear if this resilience extends to air pollution and climate change. This study uses data from the Hispanic Established Populations for the Epidemiologic Study of the Elderly (H-EPESE) linked to air pollution and climate data from the Centers for Disease Control and Prevention, to assess the association of air pollution (PM2.5 and ozone) and climate factors (drought and extreme heat) with elevated depressive symptoms. We used data from the 2005 H-EPESE which includes Mexican American adults aged 75 and older. We used generalized estimation equations to test the association between air pollution and climate factors and depressive symptoms, adjusting for sociodemographic and health characteristics. Our sample (n=1,699) was 62.0% female with a mean age of 81.6 (standard deviation: 5.0). About 19% of participants reported elevated depressive symptoms. PM2.5 concentration was associated with higher odds of depressive symptoms (odds ratio: 1.11; 95% confidence interval: 1.04, 1.19). Ozone concentration, drought, and extreme heat were not associated with depressive symptoms. Our results suggest PM2.5 is a risk factor for elevated depressive symptoms. It is unclear if older Mexican American adults are resilient to the effects of environmental risk factors relative to other demographic groups.

